# Duodenal intussusception of the remnant stomach after biliopancreatic diversion: a case report

**DOI:** 10.1186/s12893-018-0392-5

**Published:** 2018-08-14

**Authors:** J.-N. Kersebaum, C. Schafmayer, M. Ahrens, M. Laudes, T. Becker, J. H. Beckmann

**Affiliations:** 1 Klinik für Allgemeine, Viszeral-, Transplantations-, Thorax- und Kinderchirurgie, Universitätsklinikum Schleswig- Holstein, Campus Kiel, Arnold-Heller-Str. 3, 24105 Kiel, Germany; 2 Klinik für Innere Medizin I, Universitätsklinikum Schleswig- Holstein, Campus Kiel, Kiel, Germany

**Keywords:** Morbid obesity, Scopinaro, Small bowel obstruction, Intussusception

## Abstract

**Background:**

We present a rare case of an antegrade intussusception of the remnant stomach four years after a biliopancreatic diversion.

**Case presentation:**

A 55-year-old female patient presented with epigastric pain in our emergency room. Laboratory parameters showed an anemia as well as elevated transaminases and hyperbilirubinemia. The CT scan showed an intussusception of the remnant stomach into the duodenum followed by cholestasis. At laparotomy the remnant stomach was resected.

**Conclusion:**

Bowel obstruction and intussusception after bariatric surgery are a rare but often unrecognized complication. Sonography as well as a CT scan should be performed. The exploratory laparoscopy however is the most valuable diagnostic tool in patients with suspected intussusception, due to the high rate of non-specific symptoms and misinterpreted radiographic investigations.

## Backround

Bariatric surgery is a suitable treatment of morbid obesity with long term sustainable weight-loss [1, 2]. Surgical procedures are standardized, highly efficient with low rates of complications and mortality. One of the rare long term complications is small bowel obstruction [3], which can be caused by internal hernia or intussusception, with higher risks after procedures involving a Roux-en-Y reconstruction compared with sleeve gastrectomy [4, 5]. A very rare but often unrecognized problem is intussusception, most frequently seen (86%) as a retrograde intussusception of the common channel towards the jejunojejunostomy [6].

## Case presentation

The 55-year-old female patient arrived in our emergency room with epigastric for two days.

The patients’ prior events are sown on Table 1.

**Table 1:** Schematic life-line of the patient including year, weight and event

year	event	weight (kg)	weight change (kg)
1995	Vertical gastroplasty by Mason	185	− 90
2012	Gastrogastric fistula	90	
			87
2013	Biliopancreatic diversion by Scopinaro	177	
			−107
early 2017	Anemia; gastrojejunal ulcer in ogd	70	
			−3
mid 2017	Resection of remnant stomach	67	

**Fig. 1:**
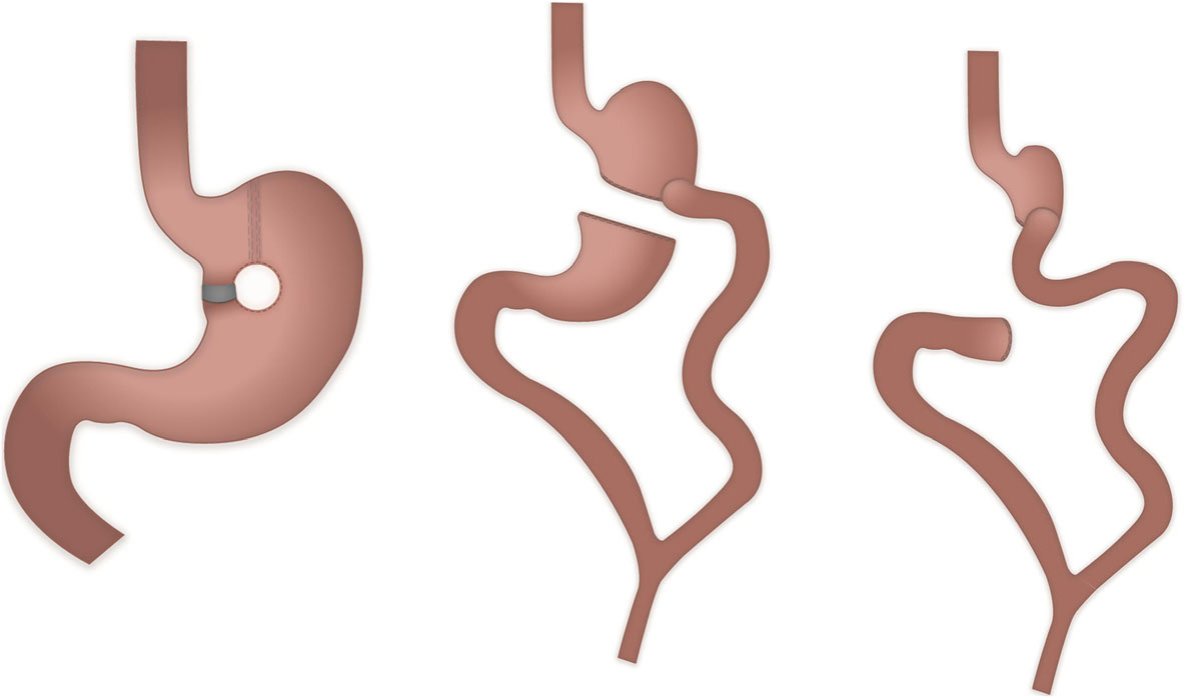
Vertical gastroplasty by Mason (left). Biliopancreatic Diversion by Scopinaro (middle). Resection of remnant stomach and redone gastrojejunal anastomosis (right)

Twenty two years before, in 1995, a vertical banded gastroplasty by Mason (Fig. 1, left) was performed (Starting weight 185 kg). After an adequate weight loss of 95 kg in the next years, a rapid weight regain of 87 kg occurred in 2013 due to a gastrogastric fistula. Therefore, the gastroplasty was converted into a biliopancreatic diversion by Scopinaro (Fig. 1, middle). The procedure was started minimal invasively, but had to be converted to open surgery, due to a short jejunal loop. The remnant stomach was not resected. Following the last surgery, an adequate weight loss of 107 kg took place. Starting in early 2017 the patient was regularly admitted to our hospital with tarry stools and iron deficiency anemia despite substitution. Esophagogastroduodenoscopies repeatedly showed a gastrojejunal anastomotic ulcer. At this point, the ulcer appeared to be the cause of the anemia. After interdisciplinary discussion the decision was made to convert the BPD to a Roux-en-Y gastric bypass with resection of the problematical gastrojejunostomy.

**Fig. 2:**
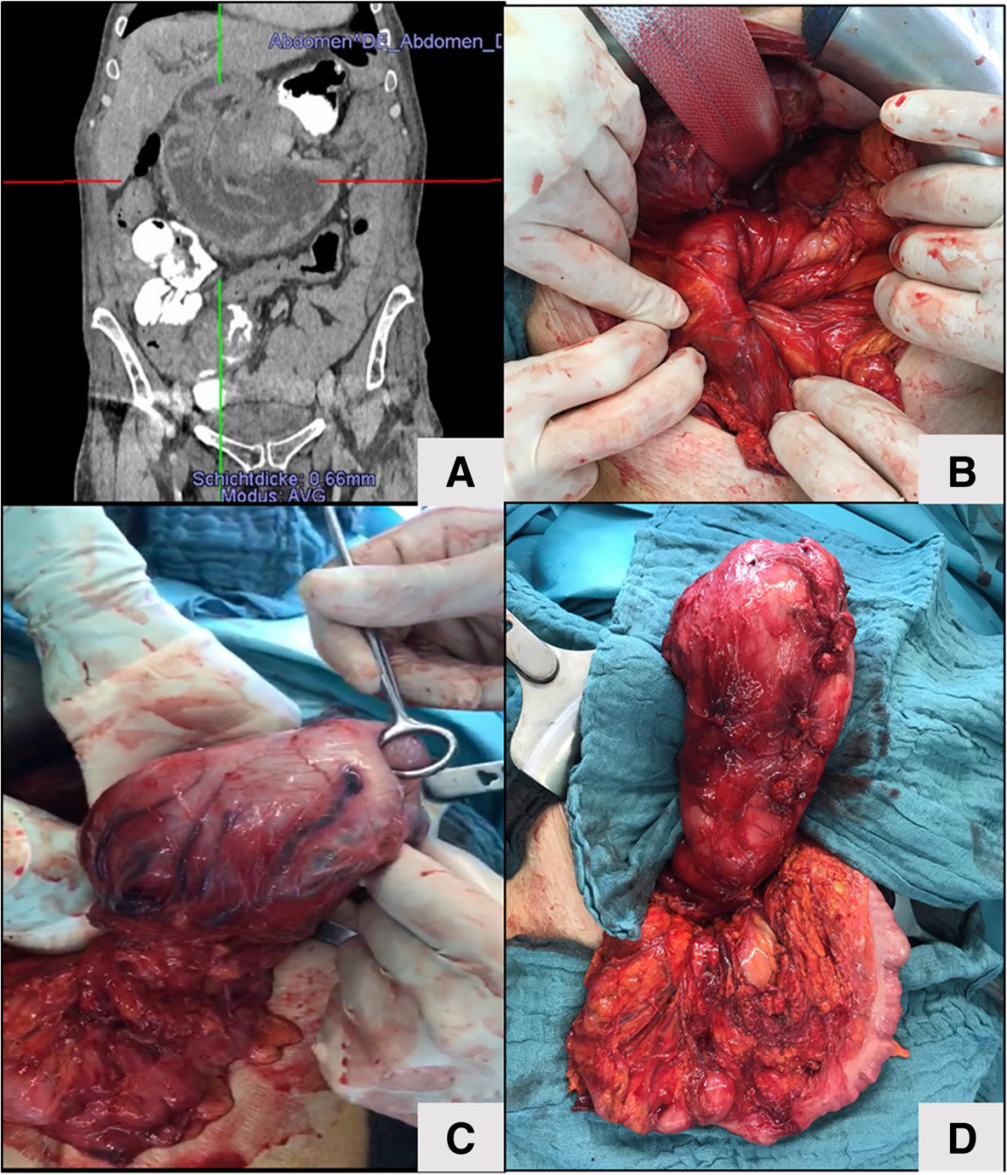
**a** Coronal cross section CT with oral contrastation with a target sign. **b** Intraoperative image of the negative lumen of the intussuscepted remnant stomach. **c** Blunt manual reposition of the remnant stomach. **d** Fully repositioned remnant stomach (top) with the transverse colon (bottom) as a size comparison

On arrival in our emergency room, the blood work showed normal leucocyte counts and a normal CRP value, but elevated transaminases and hyperbilirubinemia. An ultrasound in the emergency room showed a hyperechoic mass in the liver hilum and intrahepatic cholestasis. With the epigastric pain continuing, we decided to perform a CT-scan with oral contrastation (Fig. 1a), in which evidence was seen of an intussusception reaching the ligament of Treitz with consecutive intrahepatic cholestasis. A complete antegrade intussusception of the remnant stomach into the duodenum reaching up to the ligament of Treitz (Fig. 2b) was found during surgery. The intussusception was reduced (Fig. 2c-d) and the remnant stomach was resected (Fig. 1, right). The gastrojejunal anastomosis ulcer was resected as a short segment. A new anastomosis was fashioned using a linear stapler. The biliary as well as the common channel remained unchanged with 250 cm and 100 cm respectively. The alimentary channel was shortened to 80 cm.

Following the procedure, no further blood transfusion was needed, and the patient was discharged on the sixth day after surgery. The pathological examination showed a tumor free specimen with chronic antrum gastritis and no indication of malignancy.

## Discussion and conclusions

Intussusceptions leading to small bowel obstruction after bariatric surgery are rare. A retrospective single institution study of Zak et al. [7] showed a higher incidence of repeated operations after Roux-en-Y gastric bypass compared to sleeve gastrectomy. After six years of follow up, 6,9% of 934 patients undergoing RYGB required reoperations for other reasons than cholecystectomy. Non-healing ulcers and intussusception were responsible for 3,7% of these. In their review including 9527 patients, Koppmann et al. [3] described an overall incidence of small bowel obstruction after RYGB of 3,6%. Those complications include internal hernias (due to a Petersen’s space hernia, the mesomesenteric defect at the jejunojejunostomy and in the case of a retro colic technique, the mesocolonic defect) in ≤1% in most RYGB studies, an obstruction a the jejunojejunostomy (due to luminal narrowing or acute angulation) in 0.5% and incisional hernias in 0.3% of the cases. In only 10 reported cases included in their review, an intussusception was the cause of the small bowel obstruction. An intussusception can be retrograde or antegrade, but the retrograde intussusception of the common channel is the most common one (86%). Female gender and weight loss are risk factors for intussusception [6]. The most common symptoms are abdominal pain and/or nausea and vomiting. A peritonitis is very uncommon and only 10% of the patients have a palpable mass [8]. Compared to laboratory parameters and physical examination, imaging is much more effective method for diagnostics, with the CT being the technique of choice, with a sensitivity ranging between 64 and 81% [3, 9], but also with its limitations due to a weight limits or the radius of the gantry. An endoscopy might show a stenosis or an ulceration of the upper gastrointestinal tract, but also has its limitations, even with the double-balloon-technique, when trying to reach the remaining stomach. The exploratory laparoscopy is the most valuable diagnostic tool in patients with suspected intussusception, due to the high rate of non-specific symptoms and misinterpreted radiographic investigations. In summary, when presented with a small bowel obstruction after bariatric surgery, the surgeon has to compare the risk of an increase of the patient’s mortality in case of a bowel necrosis due to a delay in diagnostics with a relatively minimal morbidity of a negative exploratory laparoscopy.

In conclusion, agreeing with the personal guidelines presented by Bag et al. [6] and Koppmann et al. [3], we strongly emphasis the importance of early imaging diagnostics, preferably the CT and exploratory laparoscopy or even laparotomy in more complex cases.

## Abbreviations

BPD: Biliopancreatic diversion
RYGB: Roux-en Y gastric bypass
